# SAR-Efficient Sub-Volume Imaging Using Nonlinear Gradient Magnetic Fields

**DOI:** 10.3390/jimaging12060261

**Published:** 2026-06-13

**Authors:** Emre Kopanoglu, Ergin Atalar, R. Todd Constable

**Affiliations:** 1 Cardiff University Brain Research Imaging Centre (CUBRIC), School of Psychology, Cardiff University, Cardiff CF24 4HQ, UK; 2 Department of Diagnostic Radiology, Yale University, New Haven, CT 06520, USA; todd.constable@yale.edu; 3 National Magnetic Resonance Research Center (UMRAM), Bilkent University, Ankara 06800, Türkiye; ergin@ee.bilkent.edu.tr; 4 Department of Electrical and Electronics Engineering, Bilkent University, Ankara 06800, Türkiye; 5 Department of Neurosurgery, Yale University, New Haven, CT 06520, USA; 6 Department of Biomedical Engineering, Yale University, New Haven, CT 06520, USA

**Keywords:** nonlinear gradient fields, high-order gradient fields, reduced-FOV imaging, multi-dimensional selective excitation, radiofrequency pulse design

## Abstract

Excitation using nonlinear gradient magnetic fields is investigated as a means of sub-volume magnetic resonance imaging (MRI). Conventional gradient fields provide encoding along a single direction, whereas nonlinear gradient fields encode information simultaneously along at least two directions. This leads to excitation regions (FOX) that have curvilinear boundaries, which may be more tolerant to aliasing artifacts when the encoded field of view (FOV) is smaller than the FOX. This reduces the complexity of the required radiofrequency (RF) excitation pulses and enables accelerated reduced-FOV imaging with standard slice-selection RF-pulses. We demonstrate the approach using a Z2-harmonic field for cylindrical regions of interest (ROIs) with various radius/height ratios. The minimum-FOV that should be encoded is formulated in terms of ROI and RF pulse parameters to allow a theoretical evaluation of feasibility during study design. The investigated method is compared to one-dimensional and two-dimensional selective RF pulses in terms of echo time, scan time and specific absorption rate (SAR) using simulations and phantom experiments. The investigated method yields lower scan time while keeping the SAR unaltered compared to a conventional slice-selective RF pulse, and is more efficient in terms of SAR, echo time and scan time compared to two-dimensional selective excitation.

## 1. Introduction

MRI acquisitions are often long and necessitate balancing scan time against image resolution. For most applications, the region of interest is a sub-volume of the imaged subject. Imaging a smaller field of view can mean a lower number of phase-encoding steps, thereby leading to shorter scan times. Consequently, various FOV reduction techniques have been proposed.

Phase encoding steps can be reduced by localizing the excitation to conform to the ROI. Standard slice selection uses a band-limited RF pulse applied in the presence of a linear gradient field (LGF) [1,2]. When the ROI is also smaller than the cross-section, excitation may be further localized via saturation, refocusing, or multi-dimensional selective pulses [1,2,3,4,5]. Saturation and refocusing approaches use additional RF pulses before and after the excitation, respectively, while multi-dimensional selective excitation utilizes a complex RF pulse to localize the excitation to non-rectilinear volumes. However, such additional/sophisticated pulses may increase the echo and/or repetition times, especially for rapid sequences such as fast low-angle shot (FLASH) [6], and increase RF power deposition, which may become prohibitive for SAR-limited sequences. While parallel-transmit arrays can mitigate SAR [7,8], feasibility remains limited at higher field strengths due to the frequency dependence of SAR [9].

Phase encoding steps can also be reduced via parallel imaging techniques [10,11,12] by exploiting spatially varying receive-coil sensitivity profiles, and these techniques can be combined with localized excitation to reduce scan times further. Although these techniques are highly effective in regions close to the coils, the efficacy drops in regions where coil sensitivities are comparable, such as around the center of the subject [11,13].

Another approach to reduce phase encoding steps is to use actively or passively generated magnetic field inhomogeneities with nonlinear spatial variations for localization. Active methods include adjusting the region of sensitivity using spatially inhomogeneous static magnetic fields [14,15] and using surface [16,17] or volume [18] gradient coils to disturb spin phase coherence in specific regions. Passive methods use ferromagnetic materials to distort the field [19,20]. However, nonlinear field variations generally lack the spatial uniformity delivered by their linear counterparts. Moreover, steering the FOX inside the subject to conform to different ROIs may require additional fields or physical movement of the coil or the subject. Furthermore, for the same dynamic range of field values, field variations generated by higher-order fields may be lower in amplitude in certain regions. In this case, obtaining sufficient dephasing may require longer gradient pulses, thereby affecting echo/repetition times for rapid sequences.

NLGFs vary along at least two spatial directions [21], which has been used for locally improved resolution [22,23], diffusion MRI with higher gradient strength within the tissue of interest [24], improving reconstruction quality in accelerated acquisitions [25,26], excitation with reduced heating [27], and multi-dimensional excitation [28,29,30].

Because of the inherent capability of NLGFs for simultaneous multidirectional spatial encoding, a fourth approach for localizing excitation is to use nonlinear gradient fields (NLGFs) in tandem with RF pulses. A band-limited RF pulse can localize the excitation along at least two dimensions when used with an NLGF, while the localization in the presence of an LGF would only provide a single-dimensional localization. The achieved excitation profiles are curvilinear rather than rectilinear, which may be more tolerant to aliasing artifacts when the encoded FOV is smaller than the FOX and enable removal of these artifacts in post-processing. Ref. [31] used this method to image a cylindrical ROI; however, the reduction in total scan time was not explicit as it was compared to non-selective excitation of the ROI instead of one-dimensional (1D) or two-dimensional (2D) selective excitation. Furthermore, a loop coil was used to generate the NLGF, and therefore, the excitation region could not be steered inside the subject. While Refs. [21,32] used a second-order field that could be steered, the aliasing artifacts were eliminated using refocusing pulses, which affect echo time and SAR. Refs. [27,28,33,34] demonstrated multi-dimensional selective excitation with NLGFs. While replacing linear gradient fields with their nonlinear counterparts may reduce the SAR increase in multi-dimensional pulses [27,28], these approaches still lead to an overall increase in echo time and SAR, compared to the conventional slice-selective excitation.

Here, we use a second-order (Z2) field for selective excitation with a simple *sinc* RF pulse [35,36]. Similar to [31], we image cylindrical regions using a reduced FOV while allowing controlled aliasing artifacts. In contrast to prior work, the second-order field permits steering of the excitation profile using linear gradient fields (LGFs), as demonstrated in phantom experiments. We further compare the proposed approach with one-dimensional and two-dimensional selective excitation in terms of SAR, TE, TR, and the number of encoding steps—comparisons not previously reported. Unlike earlier studies [21,27,28,32,33,34], no additional or sophisticated RF waveforms are required, and therefore, SAR remains identical to conventional slice selection. We also demonstrate, both theoretically and experimentally, that the radius-to-height ratio of the cylindrical ROI can be adjusted, a capability previously suggested but not demonstrated [32]. Finally, we formulate the minimum encoded FOV in terms of the ROI height and radius, providing an explicit measure of feasibility for other applications. Using simulations and phantom experiments, we show scan time reductions of up to 81.5% relative to one-dimensional selective excitation and SAR reductions of up to 91% relative to two-dimensional selective excitation.

## 2. Materials and Methods

### 2.1. Theory

#### 2.1.1. Definitions

In this section, we derive expressions for the encoded field of view (eFOV) obtained when a simple band-limited RF pulse is applied together with a trapezoidal gradient waveform that controls either a linear or a nonlinear gradient field. Because time-limited RF pulses cannot produce ideal excitation profiles with uniform excitation inside the ROI and zero excitation outside, we adopt the following pulse-design objectives:

(i) Achieve excitation within the ROI with a predefined level of variability;
(ii) Constrain excitation in the outer volume to a specified maximum level;
(iii) Adjust the eFOV such that any aliased excitation entering the ROI remains below a specified threshold [37]. To minimize imaging time, aliasing is permitted within the eFOV but not within the ROI itself.

**Figure 1:**
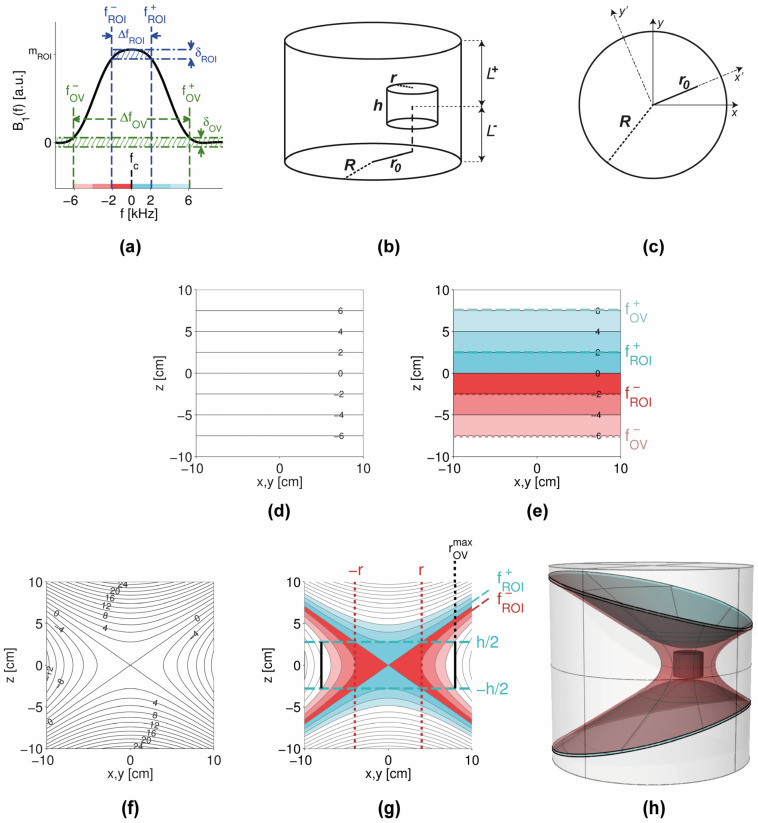
Comparison of excitation using linear and nonlinear gradient fields. (**a**) Frequency spectrum of a band-limited RF pulse with a Hanning-apodised *sinc*-envelope (time–bandwidth product: 4.25, duration: 0.54 ms). The amplitude falls to 90% of the maximum at ±2 kHz and 5% at ±6 kHz. Frequency bands are color-coded to illustrate slice selection in panels (**e**,**h**), where shades of cyan indicate positive frequencies and shades of red indicate negative frequencies compared to the central frequency. (**b**,**c**) The studied object geometry and the region of interest, illustrated from an oblique view (**b**) and a top view (**c**). (**d**,**e**) The linear z-gradient produces a linear variation in Larmor frequency (**d**), indicated by the numerical values inside the panels, relative to the central frequency. When combined with the RF pulse in panel (**a**), excitation is localized between two parallel planes (**e**). (**f**–**h**) The Z2-field generates a frequency distribution that varies in all three dimensions (**f**), indicated by the numerical values inside the panels, relative to the central frequency. When combined with the pulse in panel (**a**), excitation is confined along both longitudinal and transverse directions (**g**), yielding a cylindrically symmetric excitation profile (**h**). Panel (**h**) also illustrates an excitation profile shifted with respect to the origin, as described by Equation (6).

To simplify theoretical analysis in MRI, imaged subjects are commonly approximated as three-dimensional volumes with elliptical, circular, or rectangular cross-sections. In this work, we model the object as a circular cylinder with radius R, since a rectangular cross-section is an over-simplification whereas an elliptical cross-section introduces an extra variable without any significance on the discussion. The object is assumed to be aligned with the z-axis. The ROI inside this object is also modeled as a circular cylinder with radius r and length h, centered at a distance of $r_{0}$ from the object’s center along the direction $\overset{⃑}{r_{0}}$ (Figure 1b,c). The distances from the center of the ROI to the top and the bottom of the object (or the maximum longitudinal distances that the RF coil is sensitive to) are denoted by $L^{+}$ and $L^{-}$, respectively. Other parameters that relate to the RF pulse and the target excitation profile are demonstrated in Figure 1, and summarized below:

- $\delta_{OV}$, the maximum excitation that may alias into the ROI (OV: outer volume);
- $m_{ROI}$, target excitation inside the ROI;
- $\delta_{ROI}$, maximum excitation variation allowed inside the ROI;
- $\Delta f_{ROI}$, frequency band inside which the excitation is between $m_{ROI}\pm \delta_{ROI}$, where the corner frequencies are given by $f_{ROI}^{+}$, $f_{ROI}^{-}$;
- $\Delta f_{OV}$, frequency band outside which the excitation is between $\pm \delta_{OV}$, where the corner frequencies are given by $f_{OV}^{+}$, $f_{OV}^{-}$;
- $\zeta =\Delta f_{OV}/\Delta f_{ROI}$, bandwidth ratio of the two bands for a symmetrical frequency spectrum. For an asymmetrical frequency spectrum, the bandwidth ratio can be defined separately for positive and negative frequency bands with respect to the center (carrier) frequency ($f_{c}$): $\zeta^{+}=(f_{OV}^{+}-f_{c})/(f_{ROI}^{+}-f_{c})$ and $\zeta^{-}=(f_{c}-f_{OV}^{-})/(f_{c}-f_{ROI}^{-})$. These frequency bands are color-coded in Figure 1a and referenced in subsequent figures to facilitate visualization of excitation in the presence of nonlinear gradient fields.

For clarity, we consider isotropic spatial resolution, noting that the formulations given in the next subsections readily extend to anisotropic resolution by expressing the eFOV values in terms of the number of pixels along each direction.

#### 2.1.2. Field of View Formulation—Linear Gradient Fields

Here, we assume $L^{+}+L^{-}>2R$, reflecting typical human anatomy, and consequently, slice selection is performed along the length of the cylinder, z (Figure 1d). To obtain the target excitation level inside the ROI, $\Delta f_{ROI}$ should satisfy:

(1) $$\Delta f_{ROI}=\frac{\gamma }{2\pi }G_{z}h,$$

where $G_{z}$ is the z-gradient amplitude and γ/2π is the Gyromagnetic ratio. In this case, the encoded FOV should be:

(2) $$eFOV_{z}=\frac{{\Delta f}_{OV}+{\Delta f}_{ROI}}{2}=\frac{\left(\zeta +1\right)h}{2},$$

to allow aliasing into the eFOV, but not into the ROI (Figure 1e).

In the transverse plane, aligning one of the encoding directions with $\overset{⃑}{r_{0}}$ (Figure 1b) maximizes allowable aliasing into the eFOV while ensuring that the aliased signal remains outside the ROI. Denoting these directions using $x^{\prime }$ and $y^{\prime }$, the minimum eFOV in the transverse directions can be derived as (derivations omitted for brevity):

(3) $$eFOV_{x^{\prime }}=\sqrt{{\left(R+r\right)}^{2}-r_{0}^{2}},$$

(4) $$eFOV_{y^{\prime }}=R+r+r_{0}.$$

Readout encoding incurs negligible time cost and is therefore typically performed along the longest spatial dimension to minimize total imaging time. We denote the longest eFOV dimension as $\max\left(eFOV_{x^{\prime }},\;eFOV_{y^{\prime }},\;eFOV_{z}\right)$. For isotropic resolution, the minimum imaging time is proportional to

(5) $$t_{total}\propto \frac{eFOV_{x^{\prime }}\times eFOV_{y^{\prime }}\times eFOV_{z}}{\max\left(eFOV_{x^{\prime }},\;eFOV_{y^{\prime }},\;eFOV_{z}\right)}.$$

In Equation (5), the eFOV along the readout direction is canceled by the denominator, rendering the minimum imaging time proportional to the eFOV along the two phase-encoding directions. Note that the above formulations remain valid for ROIs shifted along z, since the encoded FOV can be shifted by changing the center frequency, $f_{c}$.

#### 2.1.3. Field of View Formulation—Nonlinear Gradient Fields

Here, we use the Z2-field, which has a three-dimensional spatial distribution characterized by $z^{2}-\left(x^{2}+y^{2}\right)/2$. The Z2-field can be steered inside the object to $\overset{⃑}{r_{0}}r_{0}=\overset{⃑}{x}x_{0}+\overset{⃑}{y}y_{0}+\overset{⃑}{z}z_{0}$ using the LGFs and a center frequency adjustment:

(6) $$\begin{array}{c} G_{z2}({\left(z-z_{0}\right)}^{2}-\frac{{\left(x-x_{0}\right)}^{2}}{2}-\frac{{\left(y-y_{0}\right)}^{2}}{2})\\ \;=G_{z2}\left(z^{2}-\frac{x^{2}}{2}-\frac{y^{2}}{2}\right)+G_{x}x+G_{y}y+G_{z}z+\frac{1}{\gamma }f_{0}, \end{array}$$

where $f_{0}=\gamma G_{z2}\left({-0.5\;x}_{0}^{2}-{0.5\;y}_{0}^{2}+z_{0}^{2}\right)$ is the Larmor frequency offset and $G_{x}=G_{z2}x_{0}$, $G_{y}=G_{z2}y_{0}$ and $G_{z}={-2G}_{z2}z_{0}$ are LGF amplitudes needed to steer the field. Consequently, the formulations that follow remain valid for regions of interest displaced from the isocenter.

The Z2-field, a cross section of which is shown in Figure 1f, vanishes on two conical surfaces defined by $z=\pm \sqrt{(x^{2}+y^{2})/2}$. Spins located between either the zero-field surface and the *z*-axis $\left(i.e.,\;\left|z\right|>\sqrt{\left(x^{2}+y^{2}\right)/2}\right)$ experience a positive frequency shift, whereas spins located between the two surfaces experience a negative frequency shift. As a result, a simple band-limited RF pulse that restricts excitation along a single direction when applied with a linear gradient (Figure 1e) instead constrains excitation along both the longitudinal and radial directions when applied with the Z2 field (Figure 1g,h).

To achieve the desired excitation within the ROI, the positive ($f_{ROI}^{+}$) and negative ($f_{ROI}^{-}$) cut-off frequencies should satisfy:

(7) $$f_{ROI}^{-}\;=-\frac{r^{2}}{2}\;\frac{\gamma G_{z2}}{2\pi },$$

(8) $$f_{ROI}^{+}=\frac{h^{2}}{4}\;\frac{\gamma G_{z2}}{2\pi }.$$

Due to the symmetry of the Z2 field distribution, the resulting excitation profile is cylindrically symmetric (Figure 1h).

Although nonlinear gradient fields enable multidirectional localization using a simple band-limited RF pulse, the excitation region exhibits extensions along the zero-field conical surfaces. Because these surfaces are obliquely oriented relative to the transverse axes, the longitudinal extent of the excitation region changes as a function of transverse position (Figure 1g).

On the other hand, using an $n^{th}$-order field (n>1) leads to $n^{th}$-order expansions/contractions in the excitation profile. For the Z2 field, the ratio of longitudinal distances along the *z*-axis corresponding to $f_{OV}^{+}$ and $f_{ROI}^{+}$ scales as $\sqrt{f_{OV}^{+}/f_{ROI}^{+}}$, compared to the linear scaling of $f_{OV}^{+}/f_{ROI}^{+}$ associated with LGFs. This effect is illustrated in Figure 1e,g, where the transition region between $f_{ROI}^{+}$ and $f_{OV}^{+}$ is contracted for the nonlinear case, providing enhanced localization.

**Figure 2:**
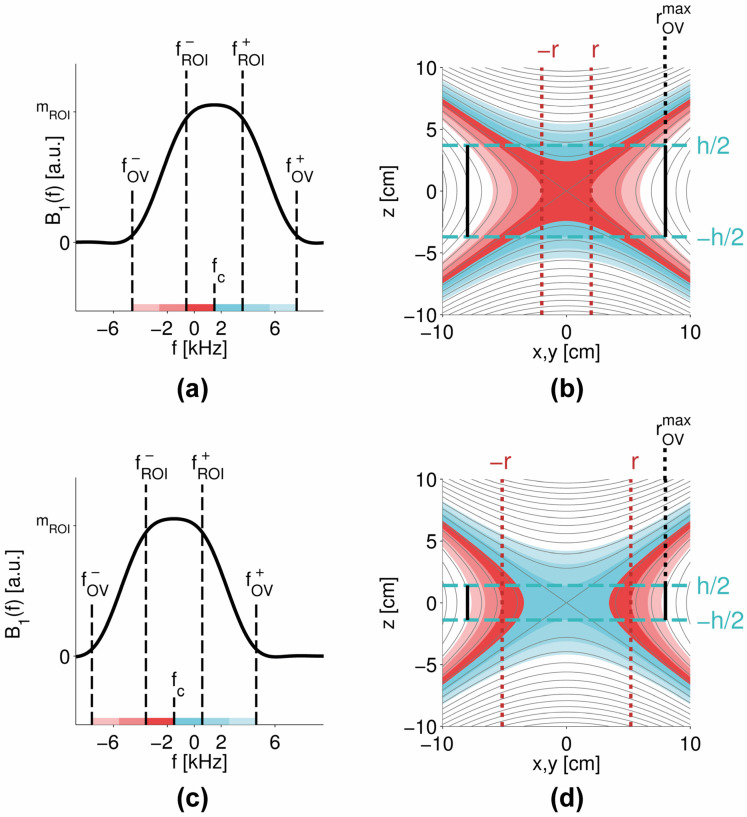
The effect of changing the center frequency on the excitation profile is demonstrated. (**a**,**b**) When the RF center frequency is increased relative to the Larmor frequency, the resulting excitation profile becomes longer and thinner, forming an elongated cylindrical region. (**c**,**d**) When the center frequency is decreased, the excitation profile becomes shorter and wider. In all cases, the excitation profiles remain cylindrically symmetric. Notably, $r_{OV}^{max}$, which denotes the maximum radial extent of the excitation region for $\left|z\right|\leq h/2$, is independent of the center frequency (see Figure 1g and Figure 2b,d). In all panels, shades of cyan indicate positive frequencies and shades of red indicate negative frequencies compared to the central frequency.

To determine the minimum encoded FOV for the NLGF case, we consider the region $\left|z\right|<h/2$ in Figure 1g. Within this region, the maximum radial distance from the z-axis where the excitation drops below $\delta_{OV}$ is denoted by $r_{OV}^{max\;}$. Using Equations (7) and (8), and $f_{c}=(f_{ROI}^{+}+f_{ROI}^{-})/2$, this distance is given by:

(9) $$r_{OV}^{max\;}=\sqrt{\left(\frac{h^{2}}{4}+\frac{r^{2}}{2}\right)\left(\zeta^{-}+1\right)},$$

which may also be expressed as:

(10) $$r_{OV}^{max\;}=\sqrt{\frac{2\pi }{\gamma G_{z}}\Delta f_{ROI}\left(\zeta^{-}+1\right)},$$

using $\Delta f_{ROI}=f_{ROI}^{+}-f_{ROI}^{-}$. Equations (9) and (10) demonstrate that the maximum radial extent of the excitation region depends only on the combined term $h^{2}+2r^{2}$, and not on the individual values of r and h. Consequently, the same RF pulse may be used to excite ROIs with different aspect ratios that share the same $h^{2}+2r^{2}$, simply by adjusting the RF center frequency. This behavior is illustrated in Figure 2, where both elongated and widened cylindrical ROIs are obtained through center-frequency modulation.

As long as the longitudinal eFOV is sufficiently large, using encoded FOV of $r_{OV}^{max\;}+r$ in both transverse directions ensures that aliasing artifacts remain outside the ROI (as will be demonstrated in Results). The transverse encoded FOV is independent of the object radius *R* unless $R<r_{OV}^{max\;}$. Accounting for both possibilities, the minimum transverse eFOVs may be written as:

(11) $$eFOV_{x}^{n}=eFOV_{y}^{n}=r+\min\left(R,r_{OV}^{max\;}\right),$$

where $\min\left(\;.\right)$ denotes the minimum of the values in parentheses. Notably, unlike the linear case (Equations (3) and (4)), the transverse eFOVs and encoding directions are independent of the ROI offset $r_{0}$ and its direction $\overset{⃑}{r_{0}}$.

The longitudinal eFOV is determined by the maximum distance from the ROI center at which the excitation around the conical surfaces falls below $\delta_{OV}$, which is given by:

(12) $$z_{OV}=\sqrt{\frac{h^{2}}{4}\frac{\left(\zeta^{+}+1\right)}{2}+\frac{r^{2}}{2}\frac{\left(\zeta^{+}-1\right)}{2}+\frac{{\left(r_{0}+R\right)}^{2}}{2}},$$

indicating that the longitudinal eFOV depends explicitly on the transverse offset $r_{0}$ (Figure 1h). The total longitudinal encoded FOV is therefore:

(13) $$eFOV_{z}^{n}=\min\left(L^{+},z_{OV}\right)+\min\left(L^{-},z_{OV}\right).$$

Finally, the readout direction is chosen as $\max\left(eFOV_{x}^{n},\;eFOV_{y}^{n},\;eFOV_{z}^{n}\right)$ and the total scan time follows from Equations (11) and (13) as:

(14) $$t_{total}^{n}\propto \frac{eFOV_{x}^{n}\times eFOV_{y}^{n}\times eFOV_{z}^{n}}{\max\left(eFOV_{x}^{n},\;eFOV_{y}^{n},\;eFOV_{z}^{n}\right)}.$$

For RF pulses with spectra symmetric about the carrier frequency $f_{c}$, the bandwidth ratios satisfy $\zeta^{+}=\zeta^{-}=\zeta$ in Equations (9), (10) and (12).

### 2.2. Investigations

Reduced-FOV imaging of cylindrical regions using the proposed approach was demonstrated through simulations and experiments, organized into the five subsections that follow.

Simulations were performed in MATLAB (R2015a, The MathWorks Inc., Natick, MA, USA). We computed the excitation generated at each voxel within the computational domain using the small-tip angle approximation [38] for both LGF and NLGF cases.

Phantom experiments were conducted on a 3T MRI system equipped with an external gradient controller and amplifier (Siemens Healthcare, Erlangen, Germany) and a Z2 gradient insert (Resonance Research Inc., Billerica, MA, USA). The peak Z2 field strength was 30 mT/m^2^. RF transmission and reception were performed using a Siemens head coil (single-channel transmit, 12-channel receive) compatible with the gradient insert. A three-dimensional gradient echo FLASH sequence (Siemens Healthcare, Erlangen, Germany) was modified to allow independent control of the Z2 gradient. Because insertion of the Z2 coil resulted in a static field ($B_{0}$) shift, the RF carrier frequency was adjusted accordingly to match the local Larmor frequency during experiments.

In all simulations and experiments, aliasing artifacts are shown to illustrate the behavior of the excitation profiles. This is not a limitation, and in practice, these can easily be masked to present just the images of the ROI to the user as the artifacts do not overlap with the ROI.

#### 2.2.1. Aliasing Artifacts

**Figure 3:**
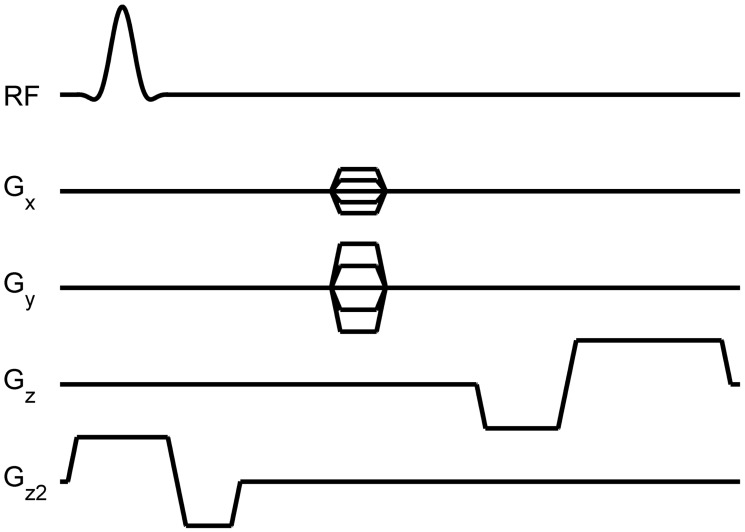
The pulse sequence used for the investigated method, which is based on a 3D gradient echo (FLASH) sequence. Slice selection is performed using the Z2-gradient, or a combination of Z2- and linear gradients when the profile is shifted with respect to the isocenter, instead of linear gradients alone. Readout encoding is performed along the z-axis to prevent aliasing artifacts.

This section demonstrates the behavior of aliasing artifacts when the Z2-field is used during excitation, and the encoded FOV is smaller than the full-FOV. Experiments were performed with a custom-made copper-sulfate-doped (1.25 g/kg) water-filled spherical phantom with a diameter of 22 cm. Simulations were performed using a numerical phantom of identical size and shape. The RF pulse had a *sinc* envelope with a time–bandwidth product (TBW) of 2.7 and a duration of 5 ms, apodised using a Hanning window (Figure 3). Simulations and experiments were conducted for three FOVs, 208×208×208 mm^3^, 64×64×208 mm^3^ and 48×48×208 mm^3^ and the resolution was 1 mm^3^, isotropic, in all cases.

#### 2.2.2. Reduced-FOV Imaging of Cylindrical Regions

The method enables imaging of cylindrical ROIs with various aspect (r/h) ratios. When $f_{c}=f_{0}$ (at the center of the Z2-field), $r/h=1/\sqrt{2}$. In one approach, r/h can be specified by calculating $f_{ROI}^{-}$, $f_{ROI}^{+}$, $\zeta^{-}$ and $\zeta^{+}$ using Equations (7)–(13), followed by designing a pulse with an asymmetric spectrum, as outlined in [37]. Alternatively, a given RF pulse can be adapted to the target aspect ratio by adjusting $f_{c}$ (Figure 2) and $G_{z2}$. This latter approach was adopted here, which preserves the RF spectral shape but introduces asymmetry about the Larmor frequency to change the aspect ratio.

In simulations, excitation profiles were visualized on the sagittal plane using a 200×200 mm^2^ FOV and a matrix size of 200×200. Experimental data were acquired using a uniform spherical phantom (18 cm diameter; Model 10496625 K2305, Siemens Healthcare) with an encoded FOV of 256×64×90 mm^2^ to demonstrate aliasing along a single direction. Additional imaging parameters were flip angle $15^{\circ }$, matrix size: 256×64×60, the number of averages: 4.

#### 2.2.3. Comparison with 1D-Selective Excitation

Although the preceding subsection illustrates flexibility in ROI geometry, it does not explicitly quantify scan time savings. Therefore, the proposed approach was compared computationally with the conventional one-dimensional (slice-selective) excitation for a range of r, h and $r_{0}$ values using Equations (5) and (14). Simulation parameters were $\zeta^{-}=\zeta^{+}=\zeta =4.5$ (i.e., TBW = 2.7), $\delta_{OV}=\delta_{ROI}=0.05$, $m_{ROI}=0.95$.

#### 2.2.4. Comparison with 2D-Selective Excitation

The proposed approach was also compared with two-dimensional selective excitation in terms of RF pulse duration, echo time (TE), repetition time (TR), scan time, and specific absorption rate (SAR). A cylindrical ROI was selected with r=15 mm, h=20 mm and $r_{0}=0$. For the investigated method, the sequence given in Figure 3 was used. Three different TBWs were used: 2.7, 5 and 8. Other design parameters were $\delta_{OV}=\delta_{ROI}=0.05$, $m_{ROI}=0.95$.

The encoded FOV was set equal to $r_{OV}^{max}$, which was calculated as 68×68 mm^2^ for TBW = 2.7, 54×54 mm^2^ for TBW = 5 and 48×48 mm^2^ for TBW = 8. For comparison, three 2D-selective RF pulses were designed to achieve the same number of encoding steps, with spatial selectivity along y and z. Pulse design followed the methods in [27,28]; using the Matching Pursuit algorithm [39], and a 2D-spoke trajectory [40]. The number of RF pulse samples was increased until the target excitation levels were obtained.

Experiments were performed for the TBW = 2.7 case using a cylindrical contrast phantom (J7239, JM Specialty Parts, San Diego, CA, USA; length 172 mm, diameter 203 mm) with 1 mm^3^ isotropic resolution. The closest available matrix size to $68^{2}$ was 64×66. Readout encoding was performed along the longitudinal axis for the proposed method and along the right–left direction for the 2D-selective pulse to minimize scan time.

For the 2D-selective experiments, the gradient insert was removed, shim currents were re-optimized, and RF transmission was performed using the body coil with reception via the standard 12-channel head coil. Although coil changes may affect B1-homogeneity, this does not influence the comparative metrics considered here. For both methods, 16 averages and a flip angle of $15^{\circ }$ were used. The RF pulse duration for the proposed method was increased to 5.1 ms to limit current in the Z2 coil and to achieve comparable TE and TR values between methods.

Because both approaches employed the same number of encoding steps, comparisons focused on pulse duration, TE, TR, scan time, and SAR. To relate linear and nonlinear gradient amplitudes, the maximum fields generated within a cylindrical volume (radius 200 mm, height 100 mm) were matched. Owing to the quadratic spatial dependence of the Z2 field, this resulted in the nonlinear gradient being approximately 20% as strong as the linear gradients at the ROI boundaries, yielding proportionally shorter LGF-only waveforms. For SAR analysis, normalized SAR was computed by scaling all RF pulses to unit duration and unit flip angle to isolate waveform-dependent effects [27].

#### 2.2.5. Steering the ROI

ROIs displaced from the isocenter were excited by applying the Z2 field in combination with appropriately scaled linear gradients. Required gradient amplitudes and RF center-frequency shifts were calculated using Equation (6). To better demonstrate the steering of the ROI, the same larger eFOV was used in all cases. The eFOV size was 256×256×160 mm^3^, the resolution (2 mm)^3^ isotropic, the flip angle $15^{\circ }$, the echo time 6.1 ms and the repetition time 12 ms.

## 3. Results

### 3.1. Aliasing Artifacts

**Figure 4:**
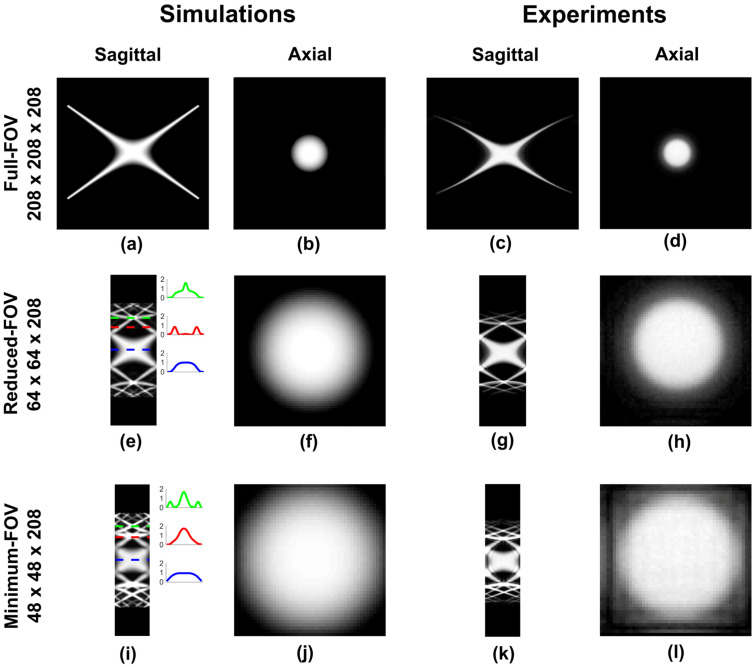
Simulated and experimental excitation profiles obtained using the Z2 field during excitation. Because the excitation profiles are cylindrically symmetric, only sagittal and axial views are shown. Reduced-FOV simulations were generated by undersampling the Fourier transform of the excitation profile. (**a**–**d**) When the full-FOV is encoded (208 mm)^3^, no aliasing occurs. In the experimental data, deviations from the simulated profiles near the FOV boundaries arise from imperfections in the Z2 field. (**e**–**h**) When the encoded FOV is reduced to 64×64×208 mm^3^, the excitation extensions along the surfaces $z=\pm \sqrt{x^{2}/2+y^{2}/2}$ fold into the encoded FOV. Because these regions extend obliquely from the origin, the central region remains free of artifacts. (**i**–**l**) Using Equation (11), the minimum FOV is calculated as 50×50×208 mm^3^. When a slightly smaller FOV of 48×48×208 mm^3^ is encoded, aliasing artifacts become visible in the axial images. Overlapping regions appear saturated because all images are normalized to the same maximum intensity defined by the central slice. Axial images in the reduced- and minimum-FOV cases are magnified for clarity. In panels (**e**,**i**), insets show the image intensity along indicated identically-colored cross-sectional lines. All images were acquired with 1 mm^3^ isotropic resolution.

The excitation profile obtained using a Z2-field resembles an hourglass geometry (Figure 1), exhibiting extensions around the surfaces $z=\pm \sqrt{{(x}^{2}+y^{2})/2}$ that reach the boundaries of the phantom (Figure 4a–d). Because these extensions are oriented obliquely to the transverse plane, reducing the encoded FOV along the transverse directions causes aliasing artifacts to fold into the outer sections of the image rather than into the region of interest (Figure 4e–l).

In this example, the encoded FOV was reduced from 208 mm to 64 mm, and then to 48 mm, along both transverse directions. The latter was selected to be slightly smaller than the minimum eFOV calculated using Equation (11) to demonstrate when aliasing artifacts become visible around the edges (Figure 4l). Because the RF pulse corresponds to a conventional slice-selection sequence, the SAR remains identical to that of a standard slice-selective sequence.

### 3.2. Reduced-FOV Imaging of Cylindrical Regions

**Figure 5:**
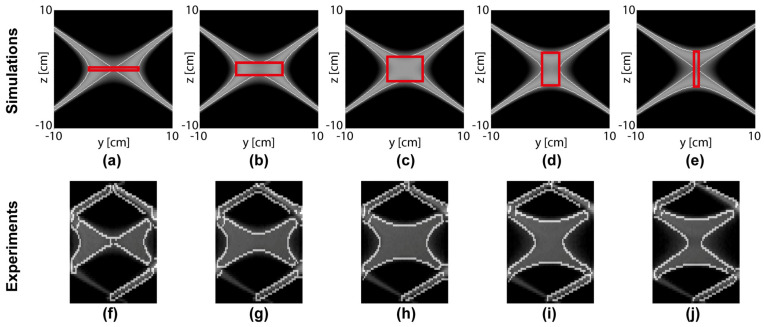
Demonstration of cylindrical ROIs with different aspect ratios (r/h) using simulations and experiments. All images are on the sagittal plane. For the simulations, the FOV is 200×200 mm^2^ whereas for the experiments, the encoded FOV is 256×64×90 mm^3^ to demonstrate the aliasing artifacts along the anterior–posterior direction (shown FOV: 128×64×90 mm^3^). The r/h ratio of the cylindrical ROI is adjusted by changing the RF center frequency. Panels (**c**,**h**) show the cylindrical region excited when the center frequency matches the Larmor frequency. When the center frequency is below the Larmor frequency, the excitation region becomes shorter and wider (**a**,**b**,**f**,**g**), whereas higher center frequencies lead to longer and thinner regions (**d**,**e**,**i**,**j**). In the simulated images, sagittal cross-sections of the cylindrical ROIs are indicated by red rectangular boxes, and contours are drawn around regions where the excitation exceeds 70% of the maximum excitation within the ROI to aid visualization of the excitation boundaries.

Figure 5 demonstrates that the aspect ratio (r/h) of the imaged cylindrical region can be controlled by adjusting the RF carrier frequency. Negative frequency offsets (i.e., $f_{c}<f_{0}$) yield shorter and wider cylindrical regions (Figure 5a,b,f,g); positive offsets lead to thinner and longer regions (Figure 5d,e,i,j). In all cases the same pulse was used, maintaining the same $h^{2}+2r^{2}$ value (Equation (10)).

### 3.3. Comparison with 1D-Selective Excitation

**Figure 6:**
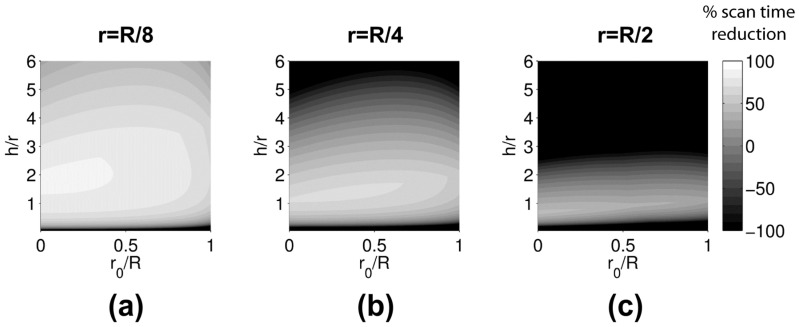
Reduction in total scan time relative to one-dimensional selective excitation for three different values of r/R. Scan time reduction was computed as $100\times (1-t_{total}^{n}/t_{total})$ using Equations (5) and (14), as functions of h/r and $r_{0}/R$. The maximum reductions were 81.5%, 63.5% and 33.5% for panels (**a**–**c**), respectively. For the linear gradient case, increasing $r_{0}$ leads to an increase in eFOV along $x^{\prime }$ and a corresponding decrease along $y^{\prime }$, resulting in the curvature of the contours around $r_{0}/R=0.5$. As the eFOV along $x^{\prime }$ increases, readout encoding along this direction may become favorable. This transition is manifested as a sharp change in the contour patterns along an implicit boundary that extends from $h/r=2,\;r_{0}/R=0$ to $h/r=4,\;r_{0}/R=1$, in panel (**a**). Similar behavior is observed in other cases.

Figure 6 compares the proposed approach with the conventional one-dimensional slice-selective excitation for imaging cylindrical ROIs within a cylindrical phantom. Maximum reductions in total scan time of 81.5%, 63.5% and 33.5% were obtained for the cases shown in Figure 6a–c, respectively. The efficiency of the proposed method increases for smaller ROIs, with larger scan time reductions observed for smaller ratios of r/R.

Importantly, the same RF pulse shape was used for both methods, and therefore these scan time reductions were achieved without any increase in normalized SAR.

Two regimes were identified in which the proposed approach becomes inefficient or infeasible. First, when r or h is sufficiently large, the $\delta_{OV}=5\%$ excitation region extends beyond the object, requiring that the whole transverse FOV is encoded, eliminating any scan time advantage (dark-shaded regions around h/r=5 in Figure 6b,c). Second, when h is very small, the ROI can be efficiently imaged using conventional slice-selection with only a few slices, making the proposed method unnecessary (dark-shaded regions below h/r=0.5 in Figure 6a–c).

### 3.4. Comparison with Two-Dimensional Selective Excitation

**Figure 7:**
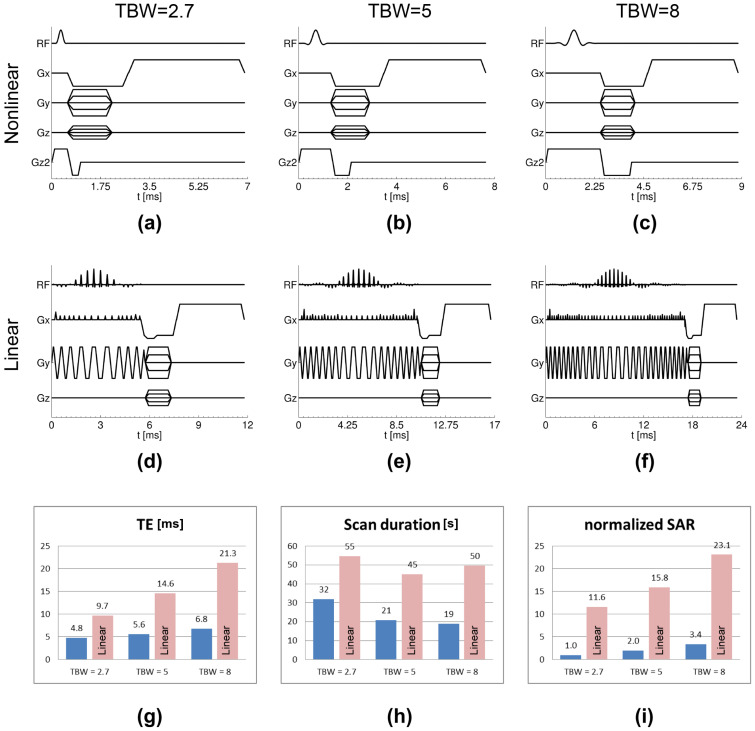
Comparison of the proposed method with two-dimensional selective excitation using linear gradient fields, in terms of RF pulse waveforms and quantitative metrics. (**a**–**c**) For the investigated method, three time–bandwidth products were used: (**a**) 2.7, (**b**) 5 and (**c**) 8. (**d**–**f**) Two-dimensional selective pulses designed to match the encoded FOV and thus the number of encoding steps of the proposed method [(**a**,**d**): 68^2^, (**b**,**e**): 52^2^, (**c**,**f**): 46^2^]. Because linear gradient fields generate stronger spatial encoding within the ROI than the nonlinear gradients, the required encoding was obtained in a shorter duration, yielding 80% shorter pulse lobes in (**d**–**f**) compared to those in (**a**–**c**), although the 2D pulses were longer overall. (**g**,**h**) The shorter pulse duration of the investigated method can be leveraged for lower echo and scan times. (**i**) Alternatively, the shorter RF pulse durations can be leveraged for reduced-FOV imaging capability with lower SAR by elongating the RF pulse duration. All RF pulses were normalized to the same flip angle and pulse duration. Normalized SAR values are reported relative to the pulse shown in (**a**).

**Figure 8:**
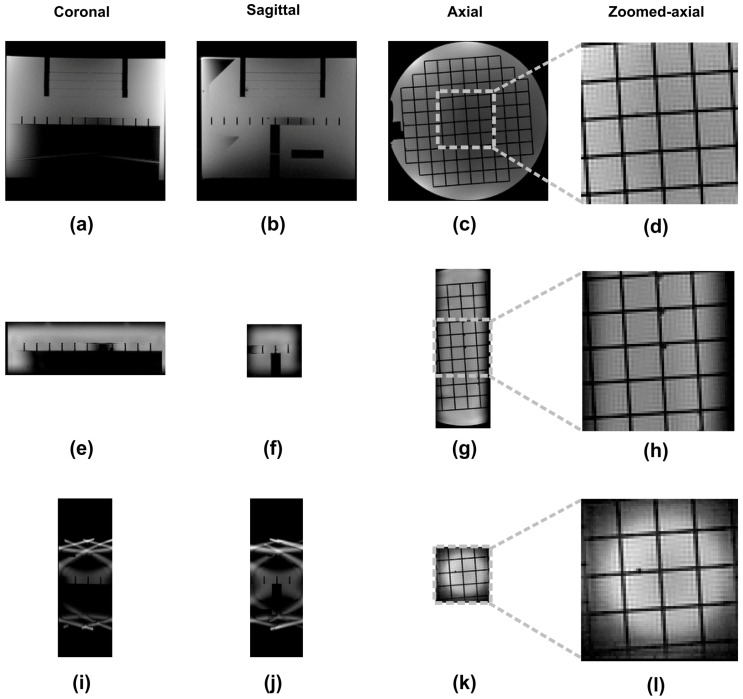
Comparison of the investigated method to two-dimensional selective excitation using linear gradient fields: experimental results. (**a**–**c**) Coronal, sagittal and axial images of the phantom (FOV: 192×192×192 mm^3^). (**d**) Magnified 64×66 mm^2^ section of the axial image. (**e**–**g**) Images obtained using the 2D-selective pulse given in Figure 7d (FOV: 192×64×66 mm^3^). (**h**) Magnified 64×66 mm^2^ section of the axial image. (**i**–**k**) Images obtained using the NLGF pulse given in Figure 6a investigated method (FOV: 64×66×192 mm^3^). The aliasing artifacts folded into the outer sections of the image, as expected. (**l**) Magnified version of the axial image. Magnified images were given for easier comparison and to show that no visible artifacts exist inside the ROI. Because the gradient insert was taken out before the 2D-pulse was applied, the alignment of the phantom was slightly different between (**e**–**h**) and (**i**–**l**).

The RF pulses used for comparison are shown in Figure 7, and the corresponding experimental results are presented in Figure 8. Because we chose to use the same maximum overall gradient field inside the whole FOV, a selection that favors the LGF-only reference pulses, the linear gradient fields are stronger within the ROI. Therefore, the pulses using only LGFs are nearly five times shorter (Figure 7d–f) than the pulses using the NLGF (Figure 7a–c). Nevertheless, the NLGF pulses are overall substantially shorter as the NLGF provides multi-dimensional rather than single-dimensional encoding. Therefore, the investigated method yields 51–68% shorter echo times, as shown in Figure 7g.

When the minimum repetition time is aimed at, these reductions in echo time directly translate into scan time savings, since both methods employ the same number of encoding steps. Comparing the minimum repetition times yielded scan time reductions of 42–62% for the investigated approach (Figure 7h).

It should be noted that echo time and repetition time comparisons depend in part on the selected gradient amplitudes. When the field amplitudes were instead scaled to yield the same maximum gradient field within the ROI rather than across the full FOV, the NLGF pulses became significantly shorter. In this case, achieved scan time reductions increase to 45–70%.

Because designed pulses have considerably different lengths, they were all normalized to the same duration and flip angle as the pulse in Figure 7a [27]. Normalized SAR values were also substantially lower for the NLGF pulses, with reductions ranging from 85% to 91% relative to the two-dimensional selective pulses considered (Figure 7i).

### 3.5. Steering the ROI

**Figure 9:**
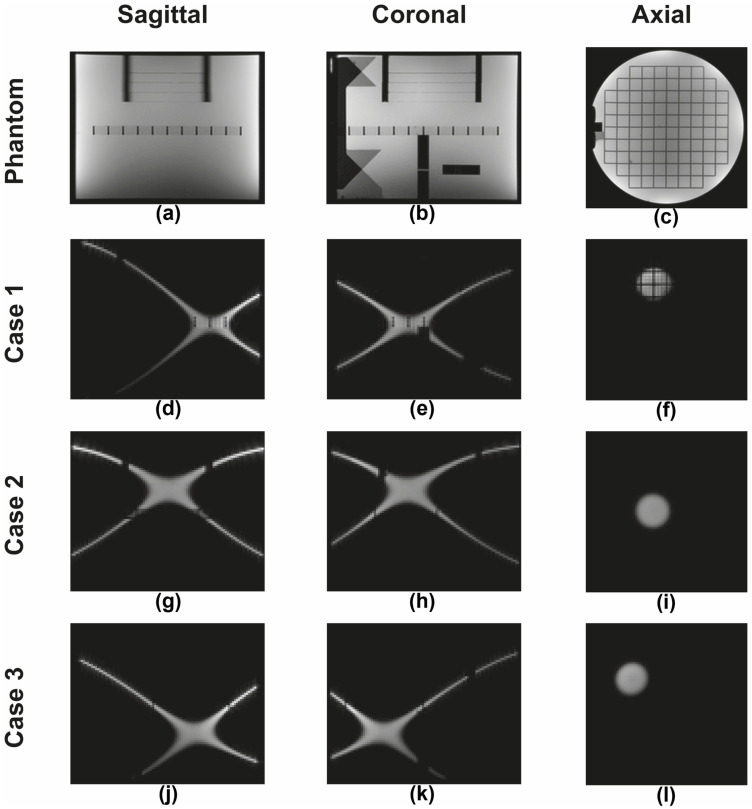
The center of the excitation region was shifted inside the phantom using LGFs and center frequency adjustments (Equation (6)). The axial, sagittal and coronal planes were shifted for each case such that the point of intersection was concentric with the center of the excitation region. The encoded FOV was 256×256×160 mm^3^, whereas the images were shown in a FOV of 200×200×160 mm^3^. Gradient echo images on the first row show structural details of the contrast phantom. Although the excitation profile was slightly distorted in certain regions, which can be seen as a slight rotation in Case 3, this is due to the generated field being imperfect (Figure 4) and not a limitation of the method. (**a**,**d**,**g**,**j**) Sagittal images. (**b**,**e**,**h**,**k**) Coronal images. (**c**,**f**,**i**,**l**) Axial images.

Experimental results in Figure 9 demonstrate that ROIs displaced from the isocenter can be successfully excited using combined linear and Z2 gradient fields. Minor distortions in the excitation profile are visible in certain regions, which is due to the imperfections in the generated field distribution (Figure 4). Nevertheless, the ROI is successfully steered to the desired location within the phantom using lower-order gradients.

## 4. Discussion

In this study, excitation using nonlinear gradient magnetic fields (NLGFs) was investigated as a strategy for sub-volume imaging. Excitation profiles generated using NLGFs have non-rectilinear geometries. Compared to conventional slice selection, these may be more forgiving of aliasing artifacts when the encoded FOV is smaller than the excitation-FOV. This property enables meaningful reductions in the number of encoding steps and, consequently, total scan time.

The primary advantage of the investigated method is that it employs the same RF pulse as conventional one-dimensional slice selection and therefore preserves specific absorption rate (SAR). We first compared the method with standard slice-selective excitation and demonstrated substantial reductions in the required number of encoding steps and, therefore, scan time. We then compared it with two-dimensional selective excitation using the same number of encoding steps, demonstrating benefits in terms of SAR, RF pulse duration, echo time, repetition time, and overall scan time.

**Selection of which nonlinear gradient field to use**

The method relies on a nonlinear gradient field that can be controlled in real time. In phantom experiments, a Z2-gradient insert and an additional control and amplifier chain were used. Nevertheless, implementing the method on a commercially available MRI system would require fewer modifications, as such systems already include nonlinear gradient fields for shimming the static magnetic field. However, immediate application is limited by the relatively weak shim amplifiers, which do not support real-time control. In Ref. [35], we used a standard commercial system and applied an offset to the Z2-shim field. Because the shim field was weak and continuously active, this demonstration necessitated an impractically long RF pulse and introduced dephasing during acquisition. However, with a stronger, real-time-controllable amplifier, the existing shim coils could be effectively utilized.

The nonlinear gradient field we used varied along all three Cartesian directions, but the encoded FOV could only be reduced in two orthogonal directions as the FOV needs to be sufficiently large along the third direction to prevent aliasing artifacts within the ROI. As a result, the method effectively provides localization in two dimensions rather than three. Similarly, a nonlinear field varying along two directions would effectively provide localization along one direction, as the encoded FOV would need to be larger than the ROI in one direction to accommodate the aliasing artifacts, effectively yielding scan times comparable to those of linear-gradient-based excitation. Consequently, nonlinear fields with three-dimensional spatial variation are better suited to the present approach. Nevertheless, not all three-dimensionally varying fields might be suitable. For the Z2-field, the profile extensions lay at an angle with respect to the transverse plane and aliased into the outer sections of the image along the longitudinal axis. For some other field distributions, the extensions might preclude similarly favorable encoding directions and make it impossible to keep the aliasing artifacts outside the ROI when linear gradients are used for encoding.

Alternatively, nonlinear gradient fields may also be used for encoding, thereby providing non-rectilinear encoding directions. Previous work has shown that combining nonlinear fields for excitation and encoding enables imaging of curved slices [41]. Analogous strategies could be used to further confine aliasing artifacts and enhance FOV-reduction capabilities.

**Shape of the region of interest**

The efficiency of sub-volume imaging depends on how closely the ROI can be matched by the excitation profile. The Z2 field is particularly well suited for exciting cylindrical ROIs whose principal axis is aligned with the *z*-direction. For obliquely oriented cylinders or other ROI geometries, efficiency may decrease when only the Z2 harmonic is available, although scan time and SAR reductions relative to one- and two-dimensional selective excitation can still be achieved. If additional second-order harmonic fields are available, the excitation profile can be rotated to target cylindrical ROIs in arbitrary orientations. For other ROI shapes, more suitable nonlinear field distributions (not necessarily second-order) can be found.

**Effect of NLGF amplitude**

Sequence timing parameters such as TE, TR, and total scan time depend in part on gradient amplitudes. Unlike linear gradients, however, nonlinear gradient fields exhibit spatially nonuniform field variations, which complicates direct specification of sequence timings in terms of gradient strength.

One approach is to maintain the maximum overall gradient field strength within the ROI across LGF and NLGF cases. Then, excitation or dephasing waveforms with NLGFs will be identical to those used with linear gradients, thereby preserving sequence timings; for example by applying dephasing waveforms simultaneously with phase-encoding gradients, as in [18].

Alternatively, the gradient field strength inside the whole volume could be limited to the same maximum. In this case, nonlinear gradient amplitudes within the ROI may be substantially lower than those generated by linear gradients (e.g., the field used in this study decays quadratically towards the isocenter), necessitating longer excitation or dephasing waveforms. This approach could increase TE, TR and scan time, but would also reduce SAR due to longer RF pulses. Here, we used this more conservative approach which led to the nonlinear gradient being only 20% as strong as the linear gradient fields inside the ROI. Fundamentally, however, the proposed approach maintains the TE, TR, and SAR of conventional slice selection.

**Specific absorption rate**

Specific absorption rate is a primary safety parameter in MRI. Although SAR limits rarely constrain sequence performance at low fields, they can limit some sequences at higher field strengths, due to the quadratic dependence of power on field strength [9]. Localizing the excitation using refocusing, saturation or multi-dimensional selective excitation pulses requires additional or more sophisticated RF pulses, which increase SAR. Excitation using parallel-transmit arrays can mitigate these increases [7,8], but purely RF-based spatial encoding is typically insufficient to achieve arbitrary localization without some SAR penalty.

In this work, the proposed approach was compared with two-dimensional selective excitation and demonstrated SAR reductions of 85–91% for the cases considered. Although SAR comparisons depend on ROI geometry and pulse-design methodology and may vary in other scenarios, these results highlight the efficiency of combining NLGFs with simple RF excitation. While fast low-angle acquisitions such as those used here are unlikely to be SAR-limited, the approach can be used with a wide range of acquisition schemes.

**RF pulse design**

Here, we employed simple RF pulses commonly used for one-dimensional selective excitation, with frequency spectra symmetric about the center frequency. In the presence of linear gradients, both sides of the RF spectrum contribute symmetrically to slice selection. For nonlinear gradient fields, however, this symmetry no longer holds. In the case of the Z2 field, excitation along the longitudinal direction is governed primarily by frequencies above the carrier frequency, whereas transverse localization is determined by frequencies below it.

Since readout encoding in this study was performed along the longitudinal direction, excitation outside the ROI along this axis was not critical. In such cases, asymmetrically designed RF spectra may be advantageous [37]. Importantly, the FOV formulations derived here (Equations (9)–(14)) remain valid for asymmetrical pulses and may therefore be incorporated into more general RF pulse-design frameworks.

**Off-resonance and Motion**

Because a simple RF pulse is used, off-resonance would cause shifting, but because of the curvilinear frequency bands, this would affect the aspect ratio of the cylindrical region that is imaged. A frequency offset that would excite a more inferior slice with LGFs would instead excite a shorter but wider region, while maintaining the $h^{2}+2r^{2}$ value.

Within-scan patient motion may affect the volume of excitation, necessitating prospective (i.e., within-scan) motion tracking and correction. This requires real-time control of lower-order fields for translations and equal- and lower-order fields for rotations. For LGFs, correction involves a weighted combination of the LGFs and a Larmor frequency adjustment, whereas when the Z2-harmonic is used, translations can be compensated by using the LGFs and a Larmor frequency adjustment (as in Equation (6)) whereas rotations would necessitate other second-order harmonics (i.e., $x^{2}-y^{2},\;xy,\;xz,\;yz$).

**Comparison with the previous literature**

Nonlinear gradient fields (NLGFs) have been widely explored for excitation and sub-volume imaging [14,15,16,17,18,19,20,21,27,28,29,32,33,34,35,36,41]. The present work builds most directly on the approaches in Refs. [21,31,32,33,34]. In [31], a loop gradient coil combined with a *sinc* RF pulse enabled reduced-FOV encoding. However, the use of a loop coil limited the ability to steer the excitation region within the subject; no formal framework was provided to define FOV boundaries; and comparisons were made against full-FOV imaging, which could also be achieved with a non-selective RF pulse, yielding even lower SAR. As a result, the scan time benefits were offset by increased SAR. Building on this prior work, we address these limitations by (i) employing a second-order field that allows flexible steering of the excitation region using standard linear gradients, (ii) formulating explicit expressions for the minimum encoded FOV to enable feasibility assessment during study design, and (iii) benchmarking against one- and two-dimensional selective excitation pulses, selected to match SAR and encoding efficiency, respectively.

Related studies using similar nonlinear fields have demonstrated suppression of excitation outside the ROI through refocusing or multidimensional RF pulses [21,32,33,34]. Our excitation profiles are consistent with those previously reported, including the ability to steer the ROI. In contrast to prior work, our focus is on preserving SAR by avoiding additional RF complexity. Furthermore, although Ref. [32] suggested that cylindrical ROIs with varying aspect ratios could be achieved using Z2 fields and frequency shifts, we provide an explicit experimental demonstration of this capability together with a systematic formulation for targeting ROIs with prescribed r, h and $r_{0}$ values. Compared to our earlier work [35,36], the present manuscript contributes a formal derivation of the minimum encoded FOV and an experimental demonstration of controllable ROI aspect ratios.

Other studies have used nonlinear gradient fields in tandem with linear gradient fields within multidimensional excitation pulses [27,28], achieving SAR reductions relative to multidimensional excitation using linear gradients alone. Nevertheless, the method investigated here still yields reduced SAR, pulse duration, TE and TR compared to multidimensional excitation.

Here, we investigated the use of nonlinear gradient fields for reduced-FOV imaging, using a Z2-field for cylindrical regions of interest. With different combinations of NLGFs, field distributions that enable imaging of sub-volumes that better conform to various anatomical structures can be obtained. This fit can be further improved by combining multidimensional selective RF pulses, parallel-transmit excitation, and nonlinear gradient fields. Finally, the investigated method can be merged with rapid acquisition approaches using multiple receive channels [10,11,12] or nonlinear gradient encoding techniques [23,41,42,43], to reduce scan time further.

## Data Availability

The data presented in this study are available in the article. Further inquiries can be directed to the corresponding author.

## Abbreviations

The following abbreviations are used in this manuscript:

MRI	Magnetic Resonance Imaging
FOV	Field of View
eFOV	Encoded Field of View
ROI	Region of Interest
FOX	Field of Excitation
TE	Echo Time
TR	Repetition Time
OV	Outer Volume
SAR	Specific Absorption Rate
FLASH	Fast Low-Angle Shot
B_0_	Main static magnetic field
B_1_	Transmit RF magnetic field
LGF	Linear Gradient Field
NLGF	Nonlinear Gradient Field
Z2 field/Z2-gradient	Second-order (quadratic) nonlinear gradient field with spatial dependence: $z^{2}-(x^{2}+y^{2})/2$
RF	Radiofrequency
TBW	Time–Bandwidth Product
1D, 2D, 3D	One-dimensional, two-dimensional, three-dimensional
